# Severity of COPD in comparison with acute myocardial infarction: evidence of in-hospital mortality in Brazil

**DOI:** 10.36416/1806-3756/e20240289

**Published:** 2025-03-18

**Authors:** Marcelo Fouad Rabahi, Guylherme Saraiva, Frederico Leon Arrabal Fernandes, Klaus Rabe

**Affiliations:** 1. Faculdade de Medicina, Universidade Federal de Goiás - UFG - Goiânia (GO) Brasil.; 2. Programa de Pós Graduação em Ciências da Saúde da UFG, Goiânia (GO) Brasil.; 3. Divisão de Pneumologia, Instituto do Coração, Hospital das Clínicas, Faculdade de Medicina, Universidade de São Paulo, São Paulo (SP) Brasil.; 4. LungenClinic Großhansdorf GmbH, Großhansdorf, Deutschland.

## TO THE EDITOR:

In 2020, COPD accounted for approximately 3.6 million deaths worldwide, (i.e., nearly 6% of all deaths occurring in that year). In addition to being a leading cause of death, COPD is associated with a burden of 79.8 million disability-adjusted life years and 14.9 million years lived with disability worldwide.^1^ ^-^ ^3^ Much of the impact of COPD is related to episodes of exacerbation, which can result in compromised health and are worsened by comorbidities that are very common in these patients. Exacerbations of COPD increase the risk of COPD-related hospitalizations and can lead to patient death from respiratory causes and cardiovascular events such as acute myocardial infarction (AMI). It has been shown that even a single moderate exacerbation over a 10-year period is associated with an increased risk of death and that when an exacerbation requires hospitalization there is a significant increase in the risk of death; this risk continues to increase in the 12 months following the exacerbation.^4^ ^-^ ^6^ Exacerbations of COPD also increase health care costs; in the United States and Canada, approximately 70-90% of all COPD-related health care costs are attributable to hospitalizations for exacerbations.^5^

Cardiovascular disease (CVD) and COPD are both major contributors to global mortality, each carrying significant individual risks. Separately, CVD has been the leading cause of global mortality since the 1960s, with a significant portion of these cases occurring in low- and middle-income countries. An estimated 17.9 million people died from CVD in 2019, accounting for 32% of all deaths in that year. In Brazil, 171,246 deaths in 2019 were attributed to coronary artery disease (CAD), corresponding to 12% of the total number of deaths in the country and 43% of all CVD deaths in the country. 

According to data from the Brazilian Unified Health Care System, the number of population-adjusted hospitalizations for AMI in the public health care system increased by 54% from 2008 to 2019.^7^ ^,^ ^8^ The incidence and impact of CAD on individual and public health justify all existing actions and campaigns focused on caring for patients with AMI. But what are the proportions of hospitalizations and in-hospital deaths among patients with AMI and those with COPD? 

We carried out an ecological study using a freely accessible database in Brazil that tabulates the care of patients hospitalized in the public health care system. We used aggregated, anonymous, and publicly available data. Our work was therefore in accordance with Brazilian National Health Council Resolution no. 674/2022 and did not require approval by a research ethics committee. 

We collected data from the Brazilian National Ministry of Health Unified Health Care System Hospital Information System in April of 2024. We searched for cases of COPD (defined in accordance with the ICD-10) using the following search terms: “bronchitis,” “emphysema,” and “other chronic obstructive pulmonary disease.” We searched for cases of AMI (defined in accordance with the ICD-10) using the search term “acute myocardial infarction.” We focused on patients > 30 years of age hospitalized in the 2019-2023 period. 

We studied hospitalization and in-hospital mortality rates for COPD and AMI separately and calculated the case-fatality rates on a year-by-year basis. We also calculated the average case-fatality rate for each disease, as well as the corresponding confidence interval. We compared the fatality rates for COPD and AMI hospitalizations using the Student’s t-test, the level of significance being set at p < 0.05. 

**Figure 1:**
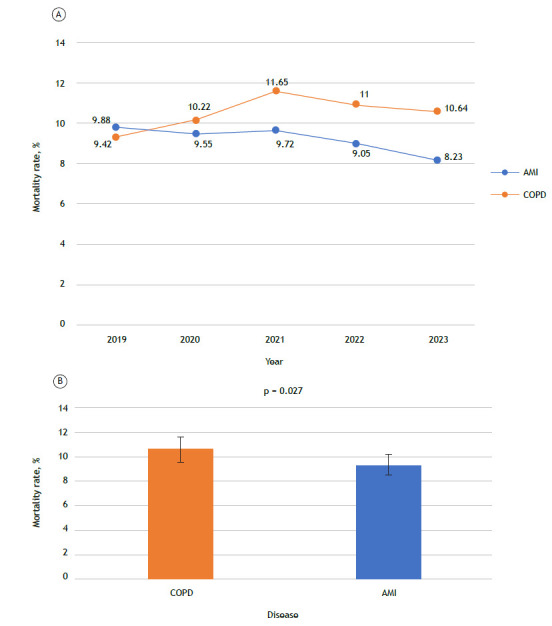
In A, mortality rates for patients > 30 years of age hospitalized for acute myocardial infarction (AMI) or COPD in the Brazilian Unified Health Care System. In B, mortality rates for patients hospitalized for COPD or AMI in the Brazilian Unified Health Care System in the 2019-2023 period. The data were compared by means of the Student’s t-test.

Of the 1,116,467 cases that we evaluated in the present study, 730,998 (65.5%) were cases of patients hospitalized for AMI and 385,469 (34.5%) were cases of patients hospitalized for COPD. Of the total number of deaths, 67,425 were due to AMI and 40,482 were due to COPD. As can be seen in Figure 1, the in-hospital fatality rates for AMI and COPD were 9.2% and 10.5%, respectively (p = 0.02). Although the difference is small, it reflects the striking differences in how COPD and AMI cases are treated. Our results highlight that being hospitalized for COPD carries a non-negligible risk of a fatal outcome. Such a risk often goes unrecognized by patients and health care teams. When disease severity and lethality are underestimated, treatment may fall short, potentially contributing to clinical deterioration and even death. 

In addition to the significant difference between fatality rates, it is important to note that these rates have increased in recent years, highlighting unmet needs in the management of COPD. Advances have been made in recent years (e.g., triple therapy), and reduced mortality rates have been noted in several large prospective studies. However, COPD mortality is still increasing worldwide.^9^

There are several barriers to the appropriate management of COPD exacerbations. Patients failing to identify their own symptoms and medical teams failing to recognize the severity of patient presentation are both likely to contribute to this issue. Barnes et al.^10^ focused on data from 14 countries, including Brazil, and noted that approximately 40% of patients take a “wait and see” approach with their worsening COPD symptoms; 56% take some action, and 4% do nothing. The symptoms of an acute coronary syndrome, however, are widely known and valued by patients and medical staff, probably as a result of continuous medical education and awareness.^10^

Our analysis reveals distinct patterns of hospitalization and in-hospital mortality in patients with COPD exacerbations and those with AMI. Although AMI is widely regarded as life-threatening, COPD exacerbations also carry a significant risk of death, which may be underrecognized. The findings of the present study underscore the need for accurate assessment of COPD exacerbations in hospital settings. 

Barriers to effective management of COPD exacerbations include delays in symptom recognition and challenges in distinguishing COPD exacerbations from other conditions with overlapping symptoms, potentially leading to inadequate treatment. Our data highlight the importance of standardized clinical guidelines to ensure proper care for COPD patients, similar to the care provided to hospitalized patients with AMI, with protocols and guidelines that can help reduce the in-hospital mortality associated with exacerbations of COPD. 
